# Supplementary material for: Compass-model physics on the hyperhoneycomb lattice in the extreme spin-orbit regime
Source: Nat Commun. 2024 Dec 6;15:10615. doi: 10.1038/s41467-024-53345-8 (PMC11624203; doi:10.1038/s41467-024-53345-8)
Supplement: Supplementary file 1 — Supplementary Information [file 41467_2024_53345_MOESM1_ESM.pdf]

Supplementary Information for
Compass-model physics on the hyperhoneycomb lattice in the extreme spin-orbit regime

Ryutaro Okuma,[1,2,*] Kylie MacFarquharson,[1] Roger D. Johnson,[3] David Voneshen,[4,5] Pascal Manuel,[4] and Radu Coldea[1]

[1] *Clarendon Laboratory, University of Oxford Physics Department, Parks Road, Oxford OX1 3PU, UK*
[2] *Institute for Solid State Physics, University of Tokyo, Kashiwa, Chiba 277-8581, Japan*
[3] *Department of Physics and Astronomy, University College London, Gower Street, London WC1E 6BT, UK*
[4] *ISIS Facility, Rutherford Appleton Laboratory, Chilton, Didcot OX11 0QX, UK*
[5] *Department of Physics, Royal Holloway University of London, Egham, TW20 0EX, UK*
(Dated: November 20, 2024)

Here we provide additional technical details on 1) sample synthesis, 2) refinement of the crystal structure from single-crystal x-ray and powder neutron diffraction, 3) magnetic structure factor calculations and magnetic structure refinement, 4) spin Hamiltonian for the hyperhoneycomb lattice, 5) mean field description of the magnetic ground state depending on various terms in the spin Hamiltonian, and 6) calculations of the spinwave spectrum and comparison with powder INS data.

Supplementary Note 1. Synthesis

Here we describe the powder synthesis of three polymorphs of Na_2PrO_3 and single crystal growth of β-Na_2PrO_3. Complementary synthesis studies focused mostly on growth of single crystals of α-Na_2PrO_3 are provided in Ref. [1]. All chemicals and samples were handled inside a nitrogen filled glovebox unless otherwise stated.

Powder synthesis of three polymorphs of Na_2PrO_3. Polycrystalline samples of α- and β-Na_2PrO_3 were synthesized by annealing cubic-Na_2PrO_3. The cubic polymorph was first synthesized by a conventional solid-state reaction of Na_2O_2 (Alfa Aesar, 95%) and Pr_6O_{11} (Merck Life Science, 99.9%). Pr_6O_{11} was calcined in air at 800°C for 24 hours. In a typical synthesis, 1.3 mmol of Pr_6O_{11} and 8.6 mmol of Na_2O_2, which amounts to 10 mol% excess use of Na_2O_2, were thoroughly ground and pressed into a pellet of ϕ = 12 mm in diameter. The pellet was loaded in an evacuated (P < 1 Pa) 40 cm long ϕ = 17 mm diameter quartz tube. The ampoule was placed in a horizontal furnace and reacted at 400°C for 48 hours. The heated sample contained purely the cubic phase, weighing 1.8 g. The heating time was determined such that all the excess Na_2O_2 is absorbed in the quartz ampouple after the reaction. The polycrystalline cubic phase sample was thoroughly ground, pressed into a pellet of ϕ = 12 mm in diameter, and loaded in an open silver tube inside the glovebox. The silver tube was sealed inside an evacuated quartz tube and reacted at 600°C and 800°C for 12 hours to obtain powder α- and β- phases, respectively.

* ryutaro.okuma@physics.ox.ac.uk

Supplementary Table I. Fractional atomic coordinates and atomic displacement parameters of β-Na_2PrO_3 deduced from single-crystal x-ray diffraction at room temperature. Atomic coordinates, and equivalent isotropic $U_{\rm eq}$ and anisotropic U_{ij} displacement parameters (in units of 10^{-3}Å^2) with estimated standard deviations in parentheses. $U_{\rm eq}$ is defined as $(U_{11} + U_{22} + U_{33})/3$. Space group: $Fddd$ (origin choice 2 at $\bar{1}$), a = 6.7641(2) Å, b = 9.7866(4) Å, c = 20.5517(6) Å, number of observed reflections = 4958, $R_{\rm int}$ = 5.88%, $R(I > 2\sigma(I))$ = 1.85%, w$R(I > 2\sigma(I))$ = 3.09%, S=0.9180. Extinction corrections were applied by fitting the raw experimental structure factor magnitudes to $|F_{\rm calc}|/\left(1 + 0.001\xi|F_{\rm calc}|^2\lambda^3/\sin 2\theta\right)^{1/4}$, where $F_{\rm calc}$ is the calculated structure factor, λ = 0.71073 Å is the x-ray wavelength and 2θ is the total scattering angle where the reflection is observed; ξ was refined to 0.00080(3).

Site	Wyckoff	x	y	z	$U_{\rm eq}$
Pr	16g	1/8	1/8	0.70879(1)	6.4(1)
Na1	16g	1/8	1/8	0.0463(1)	15.6(5)
Na2	16g	1/8	1/8	0.8796(1)	11.3(5)
O1	16e	0.8400(5)	1/8	1/8	10.0(9)
O2	32h	0.6384(5)	0.3522(3)	0.0336(1)	9.7(6)
U_{11}	U_{22}	U_{33}	U_{12}	U_{13}	U_{23}
7.9(2)	6.8(2)	4.7(2)	0.7(2)	0	0
8(1)	25(1)	13(1)	2(2)	0	0
15(1)	10(1)	9(1)	5(1)	0	0
11(2)	10(2)	9(2)	0	0	0.5(18)
10(2)	11(1)	9(1)	1(2)	0.5(12)	-0.9(11)

Single crystal growth of β-Na_2PrO_3. Single crystals of β-Na_2PrO_3 could be obtained by a solid-state reaction of Li_8PrO_6 and Na_2O as described by Wolf et al. [2]. Li_8PrO_6 was synthesized by heating stoichiometric mixture of as-received Pr_6O_{11} (Merck Life Science, 99.9%) and Li_2O (Alfa Aesar, 99.5%) in oxygen flow at 700°C for 24 hours. Li_8PrO_6 and Na_2O (Alfa Aesar, 80%) were thoroughly ground and pressed into a pellet of ϕ = 5 mm in diameter. The pellet was placed in a silver tube of ϕ = 6 mm in diameter and then sealed by flame. After heating at 700°C for several weeks, up to 0.5 mm single crystals were obtained.

Supplementary Note 2. Structural Characterization

Structural refinement from single-crystal x-ray and powder neutron diffraction. Structural information obtained from refinement of single-crystal x-ray diffraction is presented in Supplementary Table I and the quality of the agreements between data (top row) and refined model (middle row) is illustrated in Supplementary Fig. 1. Note the observed diffraction patterns show very sharp peaks with no detectable diffuse scattering, as expected for a fully-ordered crystal structure, with no indication of structural stacking faults, in contrast to the case of powder samples of the layered polymorph α-Na_2PrO_3 reported to have extensive layer stacking faults Ref. [3]. Although a lower symmetry, monoclinic $C2/c$ space group was originally proposed for β-Na_2PrO_3 in the original report in Ref. [2], our extensive single crystal x-ray diffraction data shows that the higher-symmetry orthorhombic space group $Fddd$ can describe the intensities of all observed peaks just as well, yielding an $R_{\rm int}$ of 5.88%, essentially indistinguishable from 5.74% for $C2/c$. Furthermore, the $C2/c$ model predicts several additional diffraction peaks in the $(0kl)$ and $(hk0)$ planes that are not observed in the data, compare Supplementary Fig. 1 bottom and top rows. We therefore adopt the orthorhombic structure, which is isostructural to β-Li_2IrO_3 [4]. The structural refinement performed using SHELX [5] gives a fully-ordered structure, with no site mixing and nearly isotropic U_{ij} with good agreement between the calculated and observed intensity indicated by small R factors (Supplementary Fig. 2a). The crystal structure is schematically illustrated in Supplementary Fig. 2b, all Pr sites (dark/light blue shaded spheres) are symmetry-equivalent, located inside three-fold coordinated edge-sharing O_6 octahedra, which form zigzag chains shown in dark/light blue along the $\mathbf{a} \pm \mathbf{b}$ diagonals.

Structural refinement of the neutron powder diffraction patterns at 10 K in the paramagnetic phase are shown in Supplementary Fig. 4 for detector banks that give access to different d-spacing ranges, with the resulting structural parameters listed in Supplementary Table II, the fractional coordinates are very similar with those obtained from the single-crystal x-ray refinement in Supplementary Table I. Supplementary Tables III and IV show the results of the refinement of the NaOH and Pr_6O_{11} impurity phases (2% and 1% weight phase fractions, respectively), present in the powder sample.

Ideal crystal structure. To make connection with theoretical models of spin Hamiltonians it is helpful to construct an ideal $Fddd$ structure with cubic PrO_6 octahedra. This is obtained by replacing the actual atomic fractional coordinates in Supplementary Table I by ideal coordinates as follows: Pr $\left(\frac{1}{8}\frac{1}{8}\frac{17}{24}\right)$, Na1 $\left(\frac{1}{8}\frac{1}{8}\frac{1}{24}\right)$, Na2 $\left(\frac{1}{8}\frac{1}{8}\frac{7}{8}\right)$, O1 $\left(\frac{7}{8}\frac{1}{8}\frac{1}{8}\right)$, O2 $\left(\frac{5}{8}\frac{3}{8}\frac{1}{24}\right)$, and orthorhombic unit cell lattice parameters in ratio $a:b:c = 1:\sqrt{2}:3$. In this ideal structure, by replacing Pr→Na and O1,O2→Cl, one recovers the F-centred cubic rock-salt NaCl structure oriented such that the orthorhombic unit cell axes are expressed in terms of the cubic cell axes as [202], [040], [$\bar{6}$06], with $a = 2\sqrt{2}a_0$, where a_0 is the cubic cell lattice parameter. Therefore, the actual crystal structure can be understood as a 2:1 Na:Pr cation ordering on the parent cubic rock-salt structure. The above relation to the parent cubic structure is at the origin of the (pseudo) translational symmetry of the actual $Fddd$ crystal structure by $\mathbf{c}/6$ illustrated in Supplementary Fig. 3.

Supplementary Table II. Structural parameters of β-Na_2PrO_3 obtained from the refinement of powder neutron diffraction data at 10 K. Space group: $Fddd$, $a = 6.74560(12)$ Å, $b = 9.74653(15)$ Å, $c = 20.4972(4)$ Å.

Site	Wyckoff	x	y	z	$10^3 \times U_{\rm iso}$ (Å^2)
Pr	16g	1/8	1/8	0.7087(2)	7.3(8)
Na1	16g	1/8	1/8	0.0455(3)	11.7(7)
Na2	16g	1/8	1/8	0.8788(3)	11.7(7)
O1	16e	0.8422(5)	1/8	1/8	0.4(4)
O2	32h	0.6377(4)	0.3547(1)	0.03406(6)	0.4(4)

	Bank 1	Bank 2	Bank 3	Bank 4	Bank 5
$R_{\rm Bragg}$	9.16	4.17	3.67	4.03	3.55

Supplementary Table III. Structural parameters of NaOH obtained from the refinement of powder neutron diffraction data at 10 K. Space group: $Bmmb$, $a = b = 3.3854(6)$ Å, $c = 11.377(1)$ Å.

Site	Wyckoff	x	y	z	$10^3 \times U_{\rm iso}$ (Å^2)
Na	4c	0	1/4	0.165(2)	23(13)
O	4c	0	1/4	0.365(2)	7(6)
H	4c	1/8	1/4	0.439(6)	109(13)

	Bank 1	Bank 2	Bank 3	Bank 4	Bank 5
$R_{\rm Bragg}$	4.37	6.67	9.19	9.63	10.8

Supplementary Table IV. Structural parameters of Pr_6O_{11} obtained from the refinement of powder neutron diffraction data at 10 K. Space group: $Fm\bar{3}m$, $a = b = c = 5.4567(8)$ Å. Only the lattice constants are refined because of the insufficient number of observed peaks. The occupancies of Pr and O are fixed to 1 and 11/12, respectively, as expected from the chemical formula. Isotropic atomic displacement parameters of all the atoms are fixed to $0.5/8\pi^2 = 6.3 \times 10^{-3}$Å^2.

Site	Wyckoff	x	y	z
Pr	4a	1/4	1/4	1/4
O	8c	0	0	0

	Bank 1	Bank 2	Bank 3	Bank 4	Bank 5
$R_{\rm Bragg}$	27.3	13.7	13.6	10.8	10.2

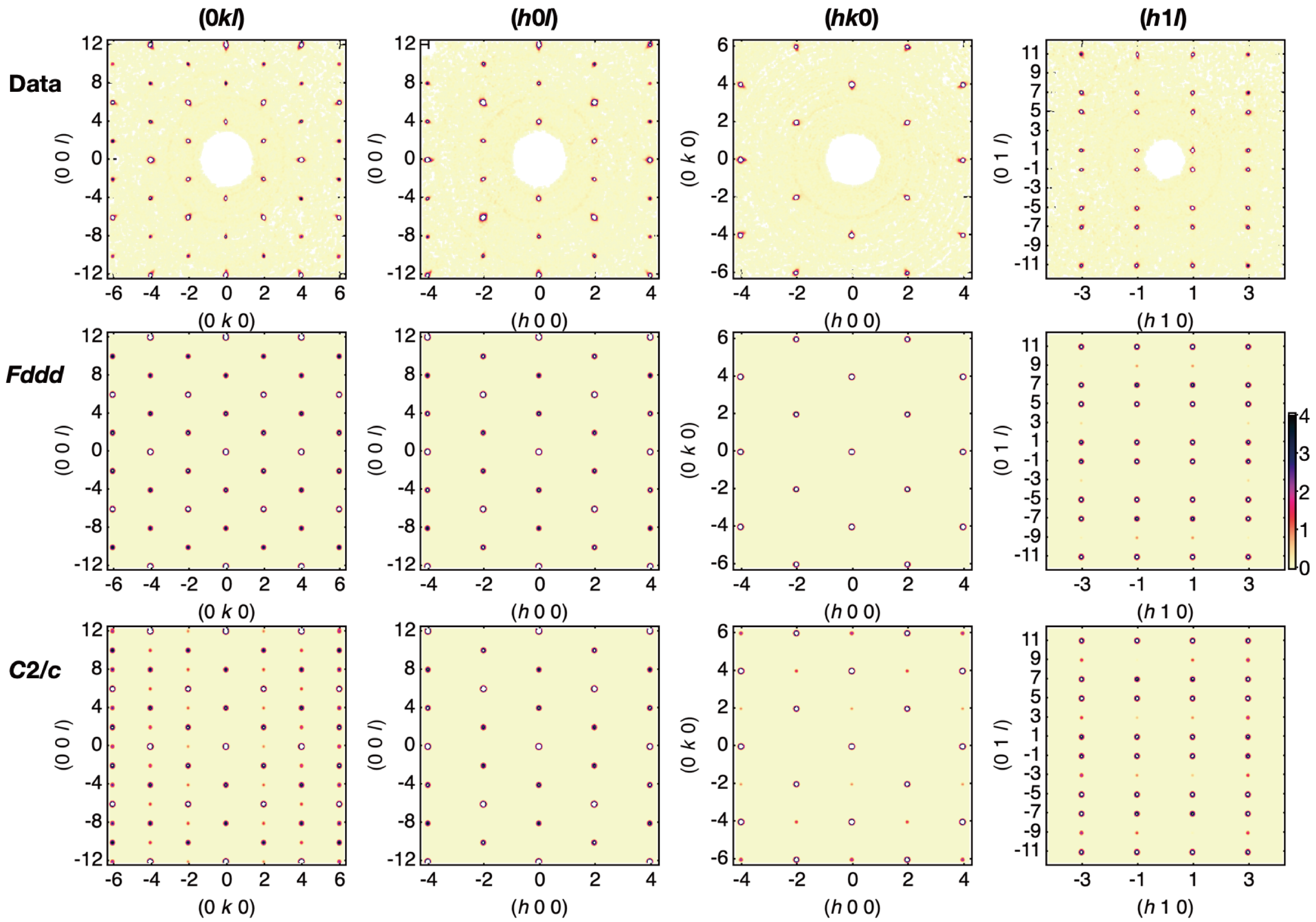

Supplementary Figure 1. Single crystal x-ray diffraction patterns in representative planes, from left to right $0kl$, $h0l$, $hk0$ and $h1l$, indexed in r.l.u. units of the orthrohorhombic $Fddd$ unit cell. Top row shows the experimentally observed diffraction intensities, middle and bottom row are calculated patterns for the best-fit $Fddd$ structural model, and the $C2/c$ model proposed by Wolf *et al.* [2], respectively. For the latter we used an idealised monoclinic unit cell with basis vectors (subscript $_{m}$) related to the orthorhombic $Fddd$ unit cell basis vectors via $\mathbf{a}_{\mathrm{m}} = \mathbf{a}$, $\mathbf{b}_{\mathrm{m}} = -\mathbf{b}$, $\mathbf{c}_{\mathrm{m}} = -(\mathbf{c}+\mathbf{a})/2$. In the $Fddd$ model, diamond glides perpendicular to the three orthorhombic axes allow only reflections with $h+k+l=4p$ (p integer) in the $0kl$, $h0l$ and $hk0$ planes. In contrast, the $C2/c$ model contains only one glide plane (perpendicular to the orthorhombic b-axis) and many peaks breaking the $h+k+l=4p$ rule are predicted in the $0kl$ and $hk0$ planes (first and third columns), none of which are observed experimentally. In the $h1l$ plane (right-most column), because of face-centring, only h odd, l odd peaks are observed, and systematic weakening of the $l=\pm3$ and ±9 peaks occurs because the crystal structure is nearly invariant by a translation by $\mathbf{c}/6$ (for details see Supplementary Fig. 3). The colour bar indicates $\log_2(1+I/255)$, where I is the diffraction intensity in arbitrary units.

Supplementary Table V. Agreement factors of the structural refinement of powder neutron diffraction data at 10 K.

	Bank 1	Bank 2	Bank 3	Bank 4	Bank 5
R_{exp}	1.02	0.30	0.22	0.21	0.20
R_{p}	15.5	11.8	8.97	9.04	8.88
R_{wp}	9.37	6.64	6.94	7.28	7.74

Supplementary Note 3. Magnetic structure factors

Magnetic structure factor. The magnetic structure factor for a magnetic Bragg peak at wavevector $\mathbf{Q}$ is

$$\boldsymbol{\mathcal{F}}(\mathbf{Q}) = f_{\mathrm{F}} \sum_n \mathbf{m}_n e^{i\mathbf{Q}\cdot\mathbf{r}_n}, \tag{1}$$

where the prefactor $f_{\mathrm{F}} = 1 + e^{i\pi(h+k)} + e^{i\pi(k+l)} + e^{i\pi(l+h)}$ is due to the F-centering of the orthorhombic structural cell. The sum extends over all sites in the primitive unit cell ($n = 1-4$), where $\mathbf{m}_n$ is the magnetic moment at site n located at position $\mathbf{r}_n$. Each of the four magnetic basis vectors has symmetry-imposed relative orientations between the $\mathbf{m}_{1-4}$ moments as listed in Supplementary Table VI, i.e. for an A-basis vector $\mathbf{m}_1 = -\mathbf{m}_2 = -\mathbf{m}_3 = \mathbf{m}_4$. Consider a magnetic structure in the basis vector combination $(A_x, \pm C_y)$ where the upper/lower sign corresponds to in-phase/out-of-phase relation between the two basis vectors, with M_x and M_y moment magnitudes ($M_{x,y}>0$) along the x and y directions for the magnetic moment at each site. The magnetic structure factor vec-

Supplementary Table VI. Fractional atomic coordinates of the Pr sites in the primitive cell, following the convention for site numbering defined in [6] for isostructural β-Li_2IrO_3. Last four columns give the definition of the magnetic basis vectors $FCAG$ described in the text. Fractional atomic coordinates xyz are with reference to the orthorhombic $Fddd$ unit cell and $z_{\rm Pr} = 0.7087(2)$ as described in Supplementary Table II.

Pr site	x	y	z	F	C	A	G
1	1/8	1/8	$z_{\rm Pr}$	1	1	1	1
2	1/8	5/8	$3/4 - z_{\rm Pr}$	1	1	-1	-1
3	3/8	3/8	$1 - z_{\rm Pr}$	1	-1	-1	1
4	3/8	7/8	$1/4 + z_{\rm Pr}$	1	-1	1	-1

Supplementary Table VII. Selection rules for the four magnetic basis vectors for all-even Miller indices and ideal Pr z-coordinate. In addition, all basis vectors contribute at all-odd indices.

Basis Vector	Reflection conditions
F	$l \neq 6p+3, h+k+l = 4p$
C	$l \neq 6p+3, h+k+l = 4p+2$
A	$l \neq 6p, h+k+l = 4p$
G	$l \neq 6p, h+k+l = 4p+2$

Supplementary Table VIII. Irreducible representations (irreps), basis vectors and magnetic space groups for $\mathbf{q} = \mathbf{0}$ magnetic structures obtained using ISODISTORT [7]. Last two columns give the basis vectors of the magnetic unit cell and origin shift in terms of the lattice basis vectors of the structural cell, i.e. for the $m\Gamma_4^-$ irrep (last row) the d' symbol in $Fd'dd$ refers to time reversal followed by a diamond glide normal to $\mathbf{b}$, i.e. mirror in the $(x0z)$ plane then translation by $\pm(\mathbf{a}+\mathbf{c})/4$.

Irrep	Basis Vectors	Magnetic Space Group	Unit Cell	Origin Shift
$m\Gamma_1^+$	G_z	$Fddd.1$	$(\mathbf{a}, \mathbf{c}, -\mathbf{b})$	$(1/4, 3/2, 1/4)$
$m\Gamma_2^+$	F_z	$Fd'd'd$	$(-\mathbf{a}, -\mathbf{b}, \mathbf{c})$	$(7/4, 7/4, 0)$
$m\Gamma_3^+$	F_x, G_y	$Fd'd'd$	$(-\mathbf{b}, -\mathbf{c}, \mathbf{a})$	$(0, 7/4, 7/4)$
$m\Gamma_4^+$	G_x, F_y	$Fd'd'd$	$(-\mathbf{a}, -\mathbf{c}, -\mathbf{b})$	$(3/2, 3/2, 3/2)$
$m\Gamma_1^-$	A_z	$Fd'd'd'$	$(\mathbf{a}, \mathbf{c}, -\mathbf{b})$	$(1/4, 3/2, 1/4)$
$m\Gamma_2^-$	C_z	$Fd'dd$	$(-\mathbf{c}, -\mathbf{b}, -\mathbf{a})$	$(3/2, 3/2, 3/2)$
$m\Gamma_3^-$	C_x, A_y	$Fd'dd$	$(-\mathbf{a}, -\mathbf{c}, -\mathbf{b})$	$(3/2, 3/2, 3/2)$
$m\Gamma_4^-$	A_x, C_y	$Fd'dd$	$(\mathbf{b}, \mathbf{c}, \mathbf{a})$	$(0, 0, 0)$

Supplementary Table IX. Structure factors for the four magnetic basis vectors, for all-odd or all-even reflections (for the ideal Pr z-coordinate).

Basis Vector	Structure Factor $\mathcal{F}(hkl)$
F	$16(-1)^l \cos(\pi l/6) \cos[\pi(h+k+l)/4]$
C	$16i(-1)^l \cos(\pi l/6) \sin[\pi(h+k+l)/4]$
A	$16i(-1)^l \sin(\pi l/6) \cos[\pi(h+k+l)/4]$
G	$-16(-1)^l \sin(\pi l/6) \sin[\pi(h+k+l)/4]$

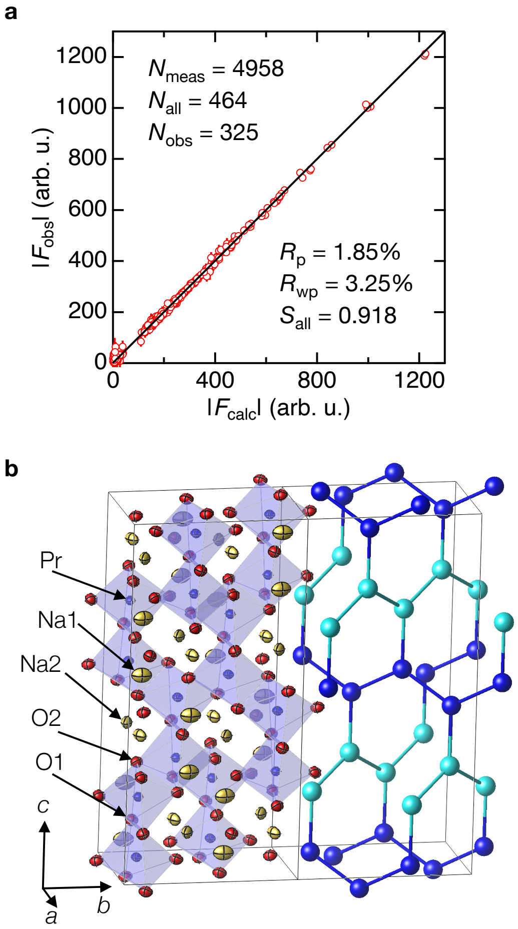

Supplementary Figure 2. **a,** Observed versus calculated x-ray structure factor magnitudes for a single crystal. $|F_{\rm obs}|$ is the experimental structure factor magnitude corrected for extinction as explained in Supplementary Table I. Error bars represent one standard deviation. Only reflections with $I > 2\sigma(I)$ defined as observed peaks are included in the refinement, but all points with non-zero intensity are included in the plot. Solid line shows the 1:1 agreement. A cluster of weak peaks ($|F_{\rm obs}| < 100$) corresponds to all-odd peaks with $l = 3 + 6p$ or to all-even peaks with $h + k + l = 4p + 2$ (p integer), which break a special reflection condition that applies to Na and Pr sites. **b,** Crystal structure obtained from the refinement of the single crystal XRD data with structural parameters listed in Supplementary Table I. Blue/red/yellow ellipsoids in the left unit cell show the anisotropic displacement parameter ellipsoids of Pr, O, and Na atoms, respectively, with black line contours along the principal planes. Right unit cell shows the hyperhoneycomb lattice formed by Pr ions, with light/dark blue colours indicating the two families of zigzag chains running along the $\mathbf{a} \mp \mathbf{b}$ basal plane diagonals.

tor in this case is $\boldsymbol{\mathcal{F}} = M_x \mathcal{F}^A \hat{\mathbf{a}} \pm M_y \mathcal{F}^C \hat{\mathbf{b}}$, where $\mathcal{F}^A$ and $\mathcal{F}^C$ are the structure factors of the A and C basis vectors given in Supplementary Table IX.

Magnetic diffraction intensity. The intensity of magnetic Bragg peaks observed in unpolarised neutron diffraction is proportional to the modulus squared of the

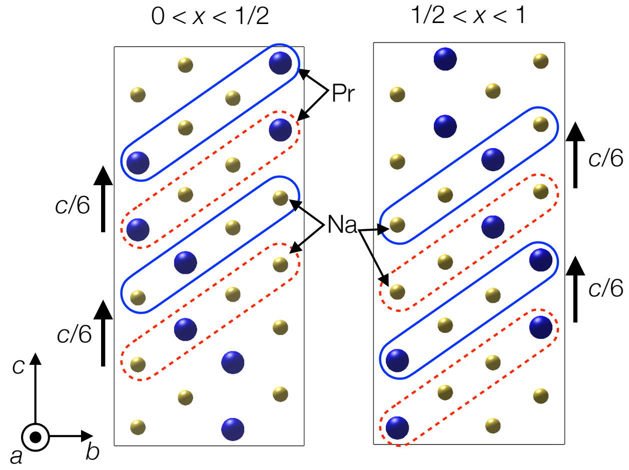

Supplementary Figure 3. Pseudo translational symmetry of the crystal structure of β-Na_2PrO_3. Projection of the atomic arrangement onto the bc plane, left for $0 < x < 1/2$ and right for $1/2 < x < 1$. Small/large spheres represent Na/Pr, Oxygens are omitted for clarity. Dashed red/solid blue outlines indicate that the full atomic arrangement can be approximately reproduced starting with half the atoms (inside dashed red outlines) and translating them by approximately $\mathbf{c}/6$ to obtain the other half of atoms (inside solid blue outlines). Destructive scattering interference between each atom and its translated pair leads to an almost exact cancellation of the structure factor for any (hkl) reflection with $l = 3 + 6p$ (p-integer). This approximate extinction rule becomes exact in the parent cubic NaCl structure.

magnetic structure factor vector perpendicular to the scattering wavevector $\mathbf{Q}$, i.e.

$$I(\mathbf{Q}) = \left(\boldsymbol{\mathcal{F}}(\mathbf{Q}) \times \hat{\mathbf{Q}}\right) \cdot \left(\boldsymbol{\mathcal{F}}^*(\mathbf{Q}) \times \hat{\mathbf{Q}}\right), \tag{2}$$

where * indicates complex conjugation and $\hat{\mathbf{Q}}$ is the unit vector along $\mathbf{Q}$. Expanding the above expression gives

$$I(\mathbf{Q}) = \left(1 - \frac{Q_x^2}{Q^2}\right) M_x^2 |\mathcal{F}^A|^2 + \left(1 - \frac{Q_y^2}{Q^2}\right) M_y^2 |\mathcal{F}^C|^2 \mp 2M_x M_y \frac{Q_x Q_y}{Q^2} \mathrm{Re}\left(\mathcal{F}^A \mathcal{F}^{C*}\right), \tag{3}$$

where Re() indicates real part. The first two terms are the respective contributions of each of the two basis vectors separately and the last term is a cross-term that is directly sensitive to the relative sign between the two basis vectors. Magnetic Bragg peaks such as (131) can be used to discriminate between the two magnetic structure models $(A_x, \pm C_y)$ as the cross-term is relatively large, obtained by direct calculation as $\mathcal{F}^A \mathcal{F}^{C*} = 32\sqrt{3} > 0$. Selecting the upper/lower sign in front of the intensity cross-term results in a lower/higher peak intensity, the difference is significant as illustrated by the solid black (lower sign)/dotted blue (upper sign) lines in Fig. 3f (left side), the lower sign $(A_x, -C_y)$ needs to be selected to correctly reproduce the observed peak intensity.

Effects of powder averaging. In a powder diffraction experiment all magnetic Bragg peaks at wavevectors $\mathbf{Q}$ related by symmetry operations of the lattice point group overlap. We have explicitly checked that all symmetry operations of the mmm lattice point group will leave the intensity expression $I(\mathbf{Q})$ invariant for all-odd reflections where both A and C basis vectors contribute. This means that all averaged peaks in the powder pattern have the same intensity and the same cross-term, so the powder diffraction data is just as sensitive to the phase difference between the two basis vectors as a single crystal experiment would be.

Symmetry of the magnetic diffraction pattern in reciprocal space. The invariance of the intensity expression $I(\mathbf{Q})$ under symmetry operations of the mmm lattice point group can also be deduced using more general symmetry considerations. The magnetic space group of the experimentally-determined magnetic structure illustrated in Supplementary Fig. 6 is $Fd'dd$ with unit cell basis vectors ($\mathbf{b}$,$\mathbf{c}$,$\mathbf{a}$), with eight primary symmetry operations $\{1, \bar{1}', 2_y, 2'_z, 2'_x, d'_y, d_z, d_x\}$, and their F-centred versions, where xyz are along the abc axes of the structural cell. Here $'$ indicates time reversal, $\bar{1}'$ is located at the origin and the middle of every zigzag bond, all 2-fold axes pass through the middle of a vertical bond such as $\left(\frac{3}{8}, \frac{3}{8}, \frac{3}{8}\right)$, d'_y is time reversal followed by a diamond glide normal to $\mathbf{b}$, i.e. mirror in $(x0z)$ plane then translation by $\pm(\mathbf{a}+\mathbf{c})/4$, and the other diamond glides also pass through the origin. The corresponding magnetic point group is $m'mm$ with symmetry operations $\{1, \bar{1}', 2_y, 2'_z, 2'_x, m'_y, m_z, m_x\}$. $I(\mathbf{Q})$ is therefore invariant under those point group operations. Furthermore, one can see by inspection of eqs. (1,2) that $I(\mathbf{Q})$ is also invariant under inversion $\bar{1}$, which maps $\mathbf{Q} \to -\mathbf{Q}$ and $\boldsymbol{\mathcal{F}}(\mathbf{Q}) \to -\boldsymbol{\mathcal{F}}(-\mathbf{Q}) = -\boldsymbol{\mathcal{F}}^*(\mathbf{Q})$ (using that $\mathbf{m}_{1-4}$ are real), so combining inversion with the above point group operations gives that $I(\mathbf{Q})$ is invariant under all symmetry operations of the paramagnetic point group $mmm1'$, which contains all operations of the mmm lattice point group. Therefore all powder-averaged magnetic reflections have the same intensity.

Magnetic domains. Since the magnetic point group $m'mm$ has half the symmetry operations of the paramagnetic point group $mmm1'$, there can be two magnetic domains, related by time reversal. Time-reversed domains have identical diffraction patterns, as time-reversal maps $\mathbf{Q} \to -\mathbf{Q}$ and $\boldsymbol{\mathcal{F}}(\mathbf{Q}) \to -\boldsymbol{\mathcal{F}}(-\mathbf{Q}) = -\boldsymbol{\mathcal{F}}^*(\mathbf{Q})$.

Magnetic structure refinement. Results of the refinement of the magnetic neutron diffraction pattern, obtained from the raw low temperature (1.4 K) pattern by subtracting the paramagnetic (10 K) pattern, is shown in Supplementary Fig. 5. Good consistency is obtained between the detector banks probing different d-spacing ranges.

Supplementary Note 4. Spin Hamiltonian

Definition of cubic xyz **axes.** For discussing the magnetic exchange it is convenient to use as reference

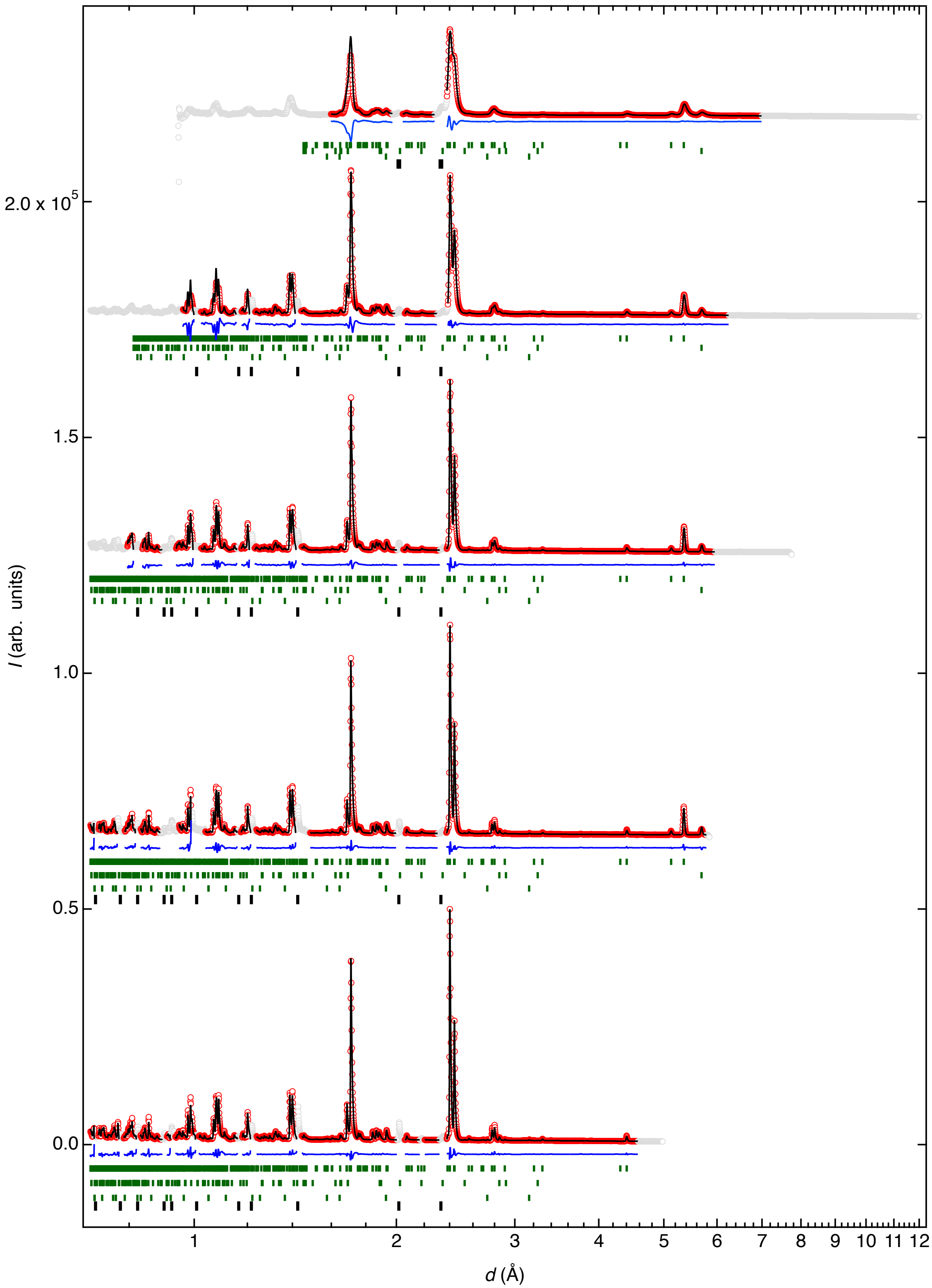

Supplementary Figure 4. Structural refinement of the neutron powder diffraction patterns measured in the paramagnetic phase at 10 K. Data sets from detector banks with different d-spacing coverage are shown vertically offset for clarity with the horizontal axis the d-spacing on a log scale. Bank number increases from 1 (top) to 5 (bottom). Red open circles, black lines and blue lines indicate measured intensity, calculated intensity and residual of the fit, respectively. The rows of thin green bars below each pattern show the Bragg peak positions of the three refined phases, nominal β-Na_2PrO_3 (topmost row), NaOH (second row) and Pr_6O_{11} (third row). The grayed out circles indicate data regions omitted from the fit, either because they are in close proximity of reflections from the aluminium sample holder (indicated by the bottom row of thick black bars under each graph), or they are in a low-resolution region of the pattern in some banks and higher-resolution data in the same d-spacing region in other banks is used instead. The agreement factors between the refinement in different banks are listed in Supplementary Table V.

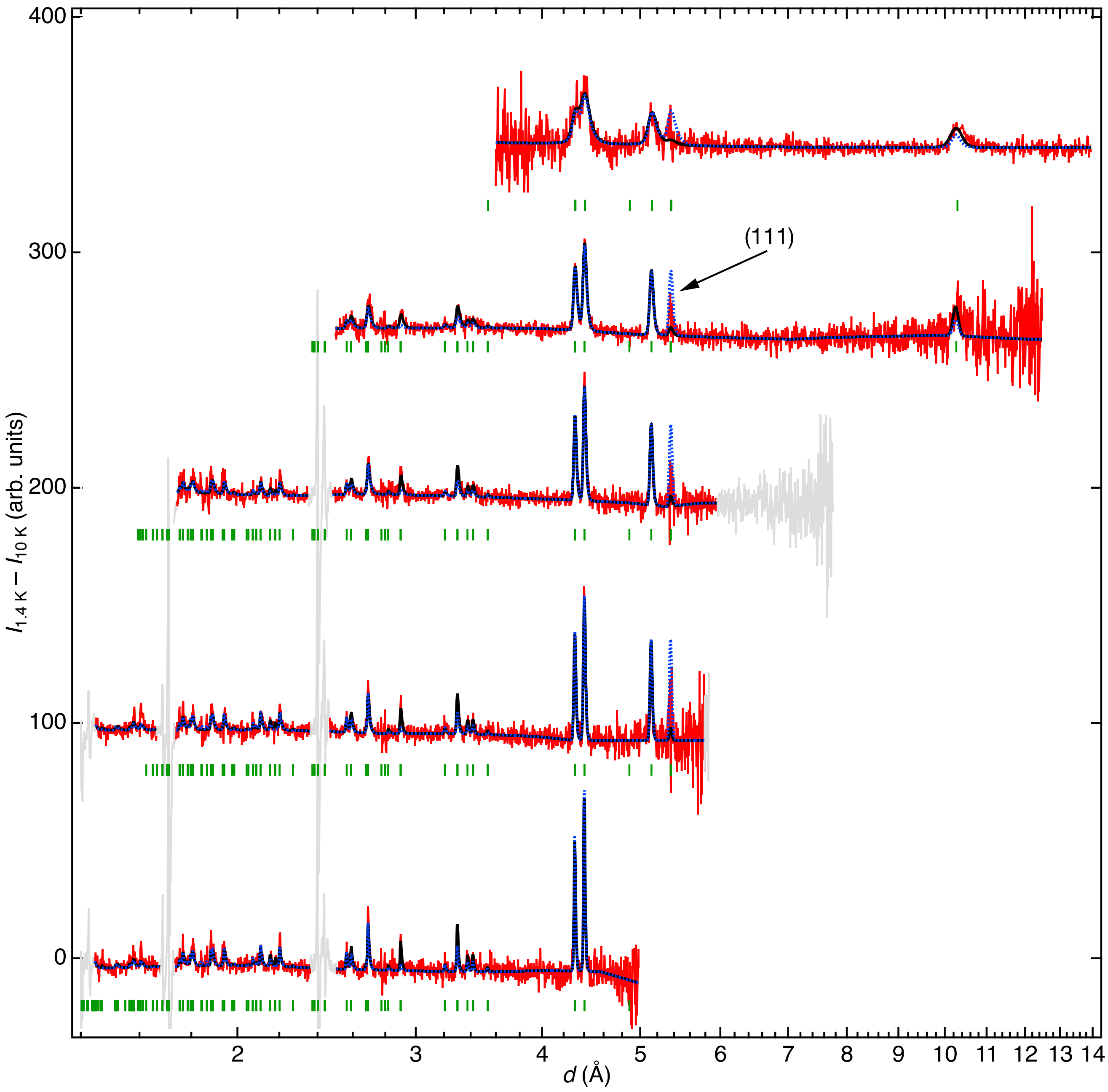

Supplementary Figure 5. Magnetic neutron powder diffraction pattern at 1.4 K obtained after subtracting from the raw data the pattern measured in the paramagnetic phase at 10 K. The vertically-offset five data sets are for the same banks as in Supplementary Fig. 4 with horizontal axis the d-spacing on a log scale. Red lines and green bars indicate the subtracted intensity and Bragg peak positions, respectively. Gray shading indicates noisy regions removed from the fit. Black/blue dotted lines are best fits to the magnetic structure models $(A_x, \mp C_y)$ described in the text. We note that the (111) peak near 5.4 Å is rather noisy because of a rather strong structural Bragg peak intensity subtracted off, so the signal there cannot be used to reliably discriminate between models. Data in all banks was refined simultaneously, the intensity scale factor in bank 2 was fixed to the value obtained from refining the structural pattern in that bank and intensity scale factors in the other banks were refined and values varied within $\sim 15\%$ between banks. Bank 2 was chosen for the intensity normalization as the structural diffraction pattern in that bank could be fitted most accurately.

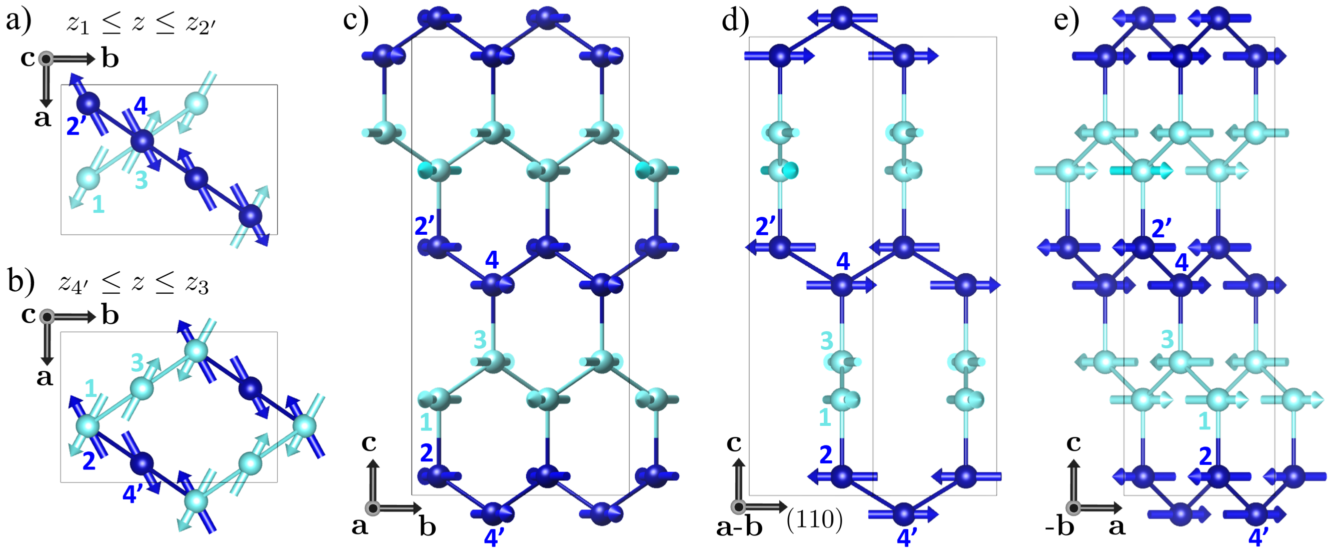

Supplementary Figure 6. Magnetic structure projected onto different crystallographic planes, from left to right ab, bc, $(110) \times \mathbf{c}$ and ac. Dark/light blue balls (arrows) indicate Pr atoms (moments) on the two families of zigzag chains running along the basal plane diagonals $\mathbf{a} \pm \mathbf{b}$, respectively. Labels 1-4 are the same as in Fig. 3g) and indicate sites equivalent to those in the primitive unit cell. $2'$ and $4'$ label sites obtained from 2 and 4 via the F-centring translation $\pm[-1/2, 0, 1/2]$, respectively. **a,b,** Projection of the magnetic structure onto the ab plane for Pr atoms with z-coordinate in the range $[z_1, z_{2'}]$ and $[z_{4'}, z_3]$, respectively, where z_i's are the z-coordinates of sites i defined above. **d,** View of the magnetic structure along the $\mathbf{a}-\mathbf{b}$ direction of the light blue zigzag chains, which emphasises that magnetic moments are oriented almost along the direction of the chain they belong to, with the two families of chains making a large angle between them as illustrated in panels **a,b**. The magnetic structure has eight primary symmetry operations $\{1, \bar{1}', 2_y, 2'_z, 2'_x, d'_y, d_z, d_x\}$, with $\bar{1}'$ located at the middle of every zigzag bond and all 2-fold axes passing through the middle of a vertical bond. d'_y is time reversal followed by a diamond glide normal to $\mathbf{b}$, i.e. mirror in $(x0z)$ plane then translation by $\pm(\mathbf{a}+\mathbf{c})/4$, and the other diamond glides also pass through the origin. The VESTA file used to plot the magnetic structure is provided in Ref. [8].

Supplementary Table X. Agreement factors of the magnetic refinement of the powder neutron diffraction data (1.4 K) to the $(A_x, -C_y)$ magnetic structure model. The refined moment sizes (in μ_B) are $M_x = 0.195(3)$ and $M_y = 0.107(4)$.

	Bank 1	Bank 2	Bank 3	Bank 4	Bank 5
R_exp	84.6	55.3	54.8	62.7	66.0
R_p	236	204	125	158	158
R_wp	71.8	48	49.2	56.8	60.1
R_Bragg	18.8	24.3	16.1	22.5	29.3

Supplementary Table XI. Agreement factors of the magnetic refinement of the powder neutron diffraction data (1.4 K) based to the (A_x, C_y) magnetic structure model. The refined moment sizes (in μ_B) are $M_x = 0.192(3)$ and $M_y = 0.079(4)$.

	Bank 1	Bank 2	Bank 3	Bank 4	Bank 5
R_exp	84.6	55.3	54.8	62.7	66
R_p	246	211	136	165	163
R_wp	77.1	50.8	53.1	59.4	63.1
R_Bragg	39.6	41.9	34.8	39.3	35.4

the ideal crystal structure with cubic PrO_6 octahedra described in Supplementary Note 2. In this case normals to the three Pr-O_2-Pr superexchange planes meeting at a site are reciprocally orthogonal and define a cubic axes frame xyz (SansSerif font to distinguish them from the orthorhombic xyz axes), with each Pr-Pr bond colour coded red/green/blue according to the axis normal to its superexchange plane. This is illustrated in Fig. 1d, where the top triad of axes shows how the cubic axes are related to the orthorhombic axes of the ideal structure following the convention introduced in Ref. [9], namely

$$\begin{pmatrix} \hat{\mathsf{x}} \\ \hat{\mathsf{y}} \\ \hat{\mathsf{z}} \end{pmatrix} = \mathcal{R} \begin{pmatrix} \hat{\mathbf{a}} \\ \hat{\mathbf{b}} \\ \hat{\mathbf{c}} \end{pmatrix} \quad \text{with} \quad \mathcal{R} = \begin{pmatrix} -\frac{1}{\sqrt{2}} & 0 & -\frac{1}{\sqrt{2}} \\ \frac{1}{\sqrt{2}} & 0 & -\frac{1}{\sqrt{2}} \\ 0 & -1 & 0 \end{pmatrix}.$$

Exchange for z-bonds. For a z-bond as illustrated in Supplementary Fig. 7, the $JK\Gamma$ Hamiltonian has the form $\mathcal{H}^{\mathsf{z}}_{ij} = J\mathbf{S}_i \cdot \mathbf{S}_j + KS^{\mathsf{z}}_i S^{\mathsf{z}}_j + \Gamma(S^{\mathsf{x}}_i S^{\mathsf{y}}_j + S^{\mathsf{y}}_i S^{\mathsf{x}}_j)$ where i and j index the two spin sites at the ends of the bond. In matrix notation, $\mathcal{H}^{\mathsf{z}}_{ij} = \mathbf{S}_i \mathcal{J}^{\mathsf{z}} \mathbf{S}^T_j$, where $\mathbf{S}_i$ is a shorthand notation for the row vector of spin components $[S^{\mathsf{x}}_i \, S^{\mathsf{y}}_i \, S^{\mathsf{z}}_i]$,

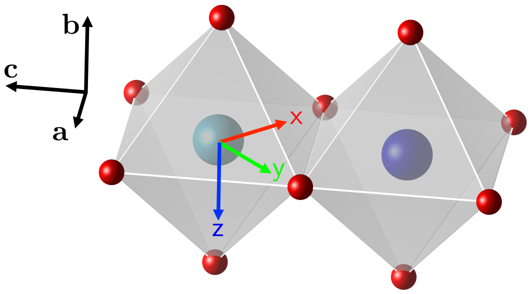

Supplementary Figure 7. Edge-sharing PrO_6 octahedra for a z bond in the ideal crystal structure showing the orientation of the xyz axes relevant to discuss the exchange Hamiltonian: z is normal to the Pr-O_2-Pr superexchange plane and x and y are along the Pr-O bonds in this plane (light/dark blue spheres are Pr, red balls are O).

^{T} is matrix transpose, and the exchange matrix is

$$\mathcal{J}^{\mathsf{z}} = \begin{pmatrix} J & \Gamma & 0 \\ \Gamma & J & 0 \\ 0 & 0 & J+K \end{pmatrix}. \quad (4)$$

In orthorhombic axes (subscript $_{abc}$) the exchange matrix is obtained as

$$\mathcal{J}^{\mathsf{z}}_{abc} = \mathcal{R}^T \mathcal{J}^{\mathsf{z}} \mathcal{R} = \begin{pmatrix} J-\Gamma & 0 & 0 \\ 0 & J+K & 0 \\ 0 & 0 & J+\Gamma \end{pmatrix}. \quad (5)$$

All (blue) z-bonds are symmetry-equivalent via F-centring translations or inversion centres located in the middle of every zigzag bond.

Energetic selection of spin orientations on z-bonds. Considering for simplicity the case of $K = 0$, $J > 0$ aligns the spins at the ends of the bond in a collinear antiparallel arrangement, and the effect of a finite Γ is to introduce an energy dependence on orientation. For $\Gamma < 0$ the directions in order of increasing energy are $\hat{x} - \hat{y}$ ($\parallel a$), $\hat{z}$ ($\parallel b$), $\hat{x} + \hat{y}$ ($\parallel c$), i.e. the energetically-preferred direction is in the exchange plane normal to the bond direction. On the other hand, for $\Gamma > 0$ the directions in order of increasing energy are $\hat{x}+\hat{y}$, $\hat{z}$, $\hat{x}-\hat{y}$, i.e. the energetically-preferred direction is along the bond direction.

Exchange for x-bonds. To discuss the exchange on x-bonds, we choose the representative (red) 1-3 bond on a $\mathbf{a} - \mathbf{b}$ chain in Fig. 3g. The $JK\Gamma$ Hamiltonian for this bond has the form $\mathcal{H}^{\mathsf{x}}_{ij} = J' \, \mathbf{S}_i \cdot \mathbf{S}_j + K' \, S^{\mathsf{x}}_i S^{\mathsf{x}}_j + \Gamma' \, (S^{\mathsf{y}}_i S^{\mathsf{z}}_j + S^{\mathsf{z}}_i S^{\mathsf{y}}_j)$ where i and j index the two spin sites at the ends of the bond. x- and z-bonds are symmetry-inequivalent, i.e. there is no symmetry operation of the crystal space group that maps one onto the other. The theoretical study of Ref. [9] considered the magnetic phase diagram under the simplifying assumption that x- and z-bonds are related by a (pseudo) 3-fold rotation around the axis normal to the plane defined by the two bonds, which however is not a symmetry operation even for the ideal structure described in Supplementary Note 2. For this simplified case $J' = J$, $K' = K$ and $\Gamma' = \Gamma$, which can be seen with reference to Fig. 3g: the (red) 1-3 x-bond is obtained from the (blue) 1-2 z-bond via rotation by $+120°$ around $\hat{\mathbf{n}}_1 = (-\hat{x} + \hat{y} - \hat{z})/\sqrt{3}$, which maps $\hat{x} \to -\hat{y}$, $\hat{y} \to -\hat{z}$ and $\hat{z} \to \hat{x}$, therefore mapping $\mathcal{H}^{\mathsf{z}}_{12} \to \mathcal{H}^{\mathsf{x}}_{13}$. However, in the following, we treat the x- and z-bonds as symmetry-inequivalent, unless explicitly stated otherwise.

In matrix notation, in the two frames the exchange matrix for the representative 1-3 x-bond is

$$\mathcal{J}^{\mathsf{x}} = \begin{pmatrix} J'+K' & 0 & 0 \\ 0 & J' & \Gamma' \\ 0 & \Gamma' & J' \end{pmatrix} \quad (6)$$

with

$$\mathcal{J}^{\mathsf{x}}_{abc} = \begin{pmatrix} J'+\frac{K'}{2} & -\frac{\Gamma'}{\sqrt{2}} & \frac{K'}{2} \\ -\frac{\Gamma'}{\sqrt{2}} & J' & \frac{\Gamma'}{\sqrt{2}} \\ \frac{K'}{2} & \frac{\Gamma'}{\sqrt{2}} & J'+\frac{K'}{2} \end{pmatrix}. \quad (7)$$

Exchange for y-bonds. The (green) y- and (red) x-bonds are symmetry equivalent. For example the y- and x-bonds sharing a common site are related by a 2-fold rotation along the z-bond sharing the same site, this rotation maps $\hat{x} \to \hat{y}$, $\hat{y} \to \hat{x}$ and $\hat{z} \to -\hat{z}$ so the interaction along the y bond emerging out of site 1 in matrix notation is

$$\mathcal{J}^{\mathsf{y}} = \begin{pmatrix} J' & 0 & -\Gamma' \\ 0 & J'+K' & 0 \\ -\Gamma' & 0 & J' \end{pmatrix}, \quad (8)$$

with

$$\mathcal{J}^{\mathsf{y}}_{abc} = \begin{pmatrix} J'+\frac{K'}{2} & -\frac{\Gamma'}{\sqrt{2}} & -\frac{K'}{2} \\ -\frac{\Gamma'}{\sqrt{2}} & J' & -\frac{\Gamma'}{\sqrt{2}} \\ -\frac{K'}{2} & -\frac{\Gamma'}{\sqrt{2}} & J'+\frac{K'}{2} \end{pmatrix}. \quad (9)$$

The exchange matrix in (8) is not simply obtained from the exchange matrix in (6) by cyclic permutation of the xyz labels, but the off-diagonal term changes sign via the symmetry operation that relates the two bonds, as noted in Ref. [9]. The off-diagonal exchange also changes sign between bonds of the same colour between chains running along the two distinct $\mathbf{a} \pm \mathbf{b}$ directions, for example in Fig. 3g between the (red) 1-3 and 2-4 x-bonds related by a 2_y axis passing through the middle of the connecting 1-2 bond. Bonds of the same colour on the same or parallel zigzag chains are identical as they are related by F-centring translations.

Energetic selection of spin orientations on x- and y-bonds. By analogy with the energetic selection discussed in the case of the z-bond in (4), for the x-bond with exchange (6) in the case $K' = 0$, $J' > 0$ and $\Gamma' > 0$, the directions in order of increasing energy are $\hat{y} + \hat{z}$, $\hat{x}$, $\hat{y} - \hat{z}$. Similarly, for the y-bond in eq. (8), the directions in order of increasing energy as $\hat{z}-\hat{x}$, $\hat{y}$, $\hat{z}+\hat{x}$. Because the

above energetically most favourable directions are different between x- and y-bonds that share a site, a compromise must be reached. The mean-field calculation developed in the following section shows that the compromise energetically-preferred direction is $-\hat{x}+\hat{y}+\sqrt{2}\hat{z}$, parallel to $\hat{\mathbf{a}}-\hat{\mathbf{b}}$, i.e. at 45° to the $\mathbf{a}$ and $\mathbf{b}$ axes. This analysis applies to the zigzag chain containing sites 1 and 3 in Fig. 3g, which is representative of zigzag chains oriented along the $\mathbf{a}-\mathbf{b}$ direction. For the zigzag chains running along the $\mathbf{a}+\mathbf{b}$ diagonal the compromise energetically-preferred direction is rotated 90° to be along $\hat{\mathbf{a}}+\hat{\mathbf{b}}$.

Supplementary Note 5. Mean-Field Model of the Magnetic Structure

Mean-field model. Starting with $J, J' > 0$ (antiferromagnetic) the magnetic structure has ordered spins collinear and antiparallel on all bonds, i.e. an A basis vector with all spin orientations degenerate. By adding an exchange $\Gamma < 0$ on the z-bonds, the a-axis becomes energetically favoured as per eq. (5), i.e. the magnetic structure becomes A_x. Inspection of eqs. (7) and (9) shows that a Γ' term couples S_x and S_y spin components, so a finite Γ' tilts the spins away from the a-axis towards b. The energetically favoured basis vector of the S_y components is C as it has antiferromagnetic alignment on both the x- and y-bonds, so both of those bonds gain energy from the Γ' exchange. The ground state becomes $(A_x, \pm C_y)$ with the mean-field energy (per site)

$$\frac{E(\phi)}{S^2/2} = (-J-2J'+\Gamma)\cos^2\phi + (J-2J')\sin^2\phi \pm 2\sqrt{2}\Gamma'\sin\phi\cos\phi$$

with $\phi > 0$ the tilt angle of the spins away from the a-axis and the upper (lower) sign in the ground state basis vectors combination and the energy expression chosen for $\Gamma' < 0$ ($\Gamma' > 0$). Minimising $E(\phi)$ gives the equilibrium tilt angle ϕ in terms of exchanges as

$$\tan 2\phi = \frac{2\sqrt{2}|\Gamma'|}{2J-\Gamma}, \tag{10}$$

and the minimum energy

$$\frac{E_0}{S^2/2} = -2J' + \frac{\Gamma}{2} - \sqrt{\left(J-\frac{\Gamma}{2}\right)^2 + 2\Gamma'^2}. \tag{11}$$

Setting $\Gamma' = 0$ gives $\phi = 0$ and $E_0 = (-J-2J'+\Gamma)S^2/2$, i.e. recovers the pure A_x magnetic structure selected by $J, J' > 0$ and $\Gamma < 0$. On the other hand, setting $\Gamma = 0$ and $\Gamma' > 0$ makes the experimentally determined magnetic structure $(A_x, -C_y)$ with $M_y/M_x = \tan\phi$, degenerate with another structure $(C_x, -A_y)$ with $M_x/M_y = \tan\phi$, with $\tan 2\phi = \sqrt{2}\Gamma'/J$ in both cases.

Non-collinear order from frustration of Γ and Γ' exchanges. The physical interpretation of $\Gamma < 0$ is that antiferromagnetically-aligned spins on the z-bonds prefer to be in the ab plane, closest to the a-axis. $\Gamma' > 0$ on the x- and y-bonds means that antiferromagnetically aligned spins prefer to be also in the ab plane, but closest to the $\hat{\mathbf{a}} \pm \hat{\mathbf{b}}$ directions, for the zigzag chains running along the $\mathbf{a} \pm \mathbf{b}$, respectively, as we show below. Those energetically most favourable spin directions are mutually incompatible, with the consequence that a compromise is reached. Starting with $\Gamma < 0$ and $\Gamma' = 0$ the magnetic structure is A_x with spins along a as preferred by the z-bonds. Switching on $\Gamma' > 0$ rotates the spins at the ends of each z-bond around the bond axis, in opposite senses for the two ends such as to bring the spins closer to the directions preferred by the two different types of zigzag chains at the bond ends. The above spin rotations keep the spins on the zigzag chains antiparallel, which maximises the energy gain from the J' exchange. Through those spin rotations both x- and y-bonds gain energy via Γ', more than the energy lost on the z bonds due to spins at the two ends rotating away from the optimal antiparallel alignment along a favoured by Γ. Focusing on the 1-2 z-bond in Fig. 3g, upon switching on $\Gamma' > 0$ the spin on site 1 rotates away from $\mathbf{a}$ by an angle ϕ towards $\hat{\mathbf{a}}-\hat{\mathbf{b}}$, whereas spin 2 rotates by the same angle away from $-\mathbf{a}$ towards the $-(\mathbf{a}+\mathbf{b})$ direction. According to eq. (10), the tilt angle ϕ increases monotonically from 0 upon increasing Γ' and eventually reaches the maximum value $\pi/4$ in the limit of large $\Gamma' \gg J, -\Gamma$. In this limit the zigzag chains are effectively decoupled, and each chain has collinear antiferromagnetic order along its own preferred direction, obtained from $\phi = \pi/4$ as $\hat{\mathbf{a}} \pm \hat{\mathbf{b}}$ for chains running along the $\mathbf{a} \pm \mathbf{b}$ directions.

Relation to the phase diagram of the $JK\Gamma$ model. We note that the experimentally-determined magnetic structure is not contained in the magnetic phase diagram of the $JK\Gamma$ model under the simplifying assumption that all bonds are symmetry-equivalent, i.e. $J' = J$, $K' = K$ and $\Gamma' = \Gamma$. In particular, in this model $J > 0$ and $\Gamma < 0$ select the in-phase (A_x, C_y) ground state (labelled AF_a in Ref. [9]), the out-of-phase ground state $(A_x, -C_y)$ can only be obtained in a form where the dominant basis vector is C_y, not A_x (structure labelled SS_b in Ref. [9]) in a region in parameter space where $-K \geq J > 0$ and $\Gamma > 0$; the powder-averaged spinwave spectrum for a representative set of exchange values from that region of parameter space is shown in Supplementary Fig. 8, which differs qualitatively from the experimentally observed spectrum in Fig. 4a.

Comparison to a Heisenberg model with Dzyaloshinskii Moriya (DM) interactions. For completeness we note that a non-collinear magnetic structure could in principle also be stabilized by an antiferromagnetic Heisenberg exchange J on all nearest-neighbour bonds and a symmetry-allowed DM interaction on the z-bonds, $\mathbf{D}\cdot(\mathbf{S}_i \times \mathbf{S}_j)$ where $\mathbf{D} \parallel \mathbf{c}$ and i, j index the lower/upper sites on all z-bonds, which are 2-fold rotation axes of the crystal structure. However, to quantitatively reproduce the experimentally deduced non-collinearity would require $D/J = \tan 2\phi \simeq 1.5$ (using the experimentally determined $\phi = \arctan(M_y/M_x)$

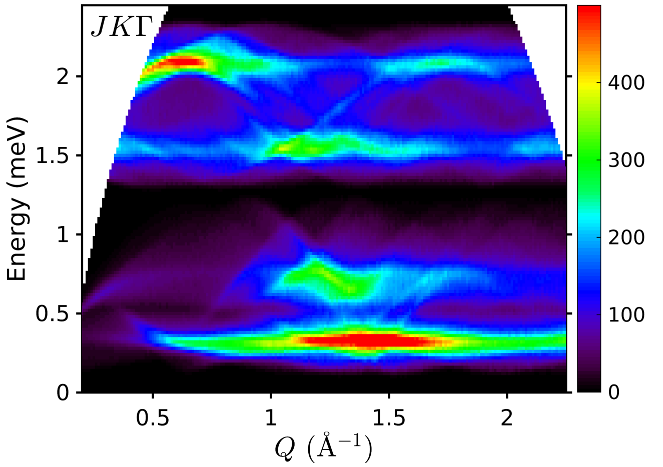

Supplementary Figure 8. Powder-averaged spinwave spectrum for the $JK\Gamma$ Hamiltonian for which the spectrum along a high-symmetry path in reciprocal space is plotted in Supplementary Fig. 10e. The calculation includes magnetic form factor, neutron polarization and convolution with the estimated experimental energy resolution and should be compared to the experimental data in Fig. 4a. The colour bar indicates the calculated scattering intensity in arbitrary units on a linear scale.

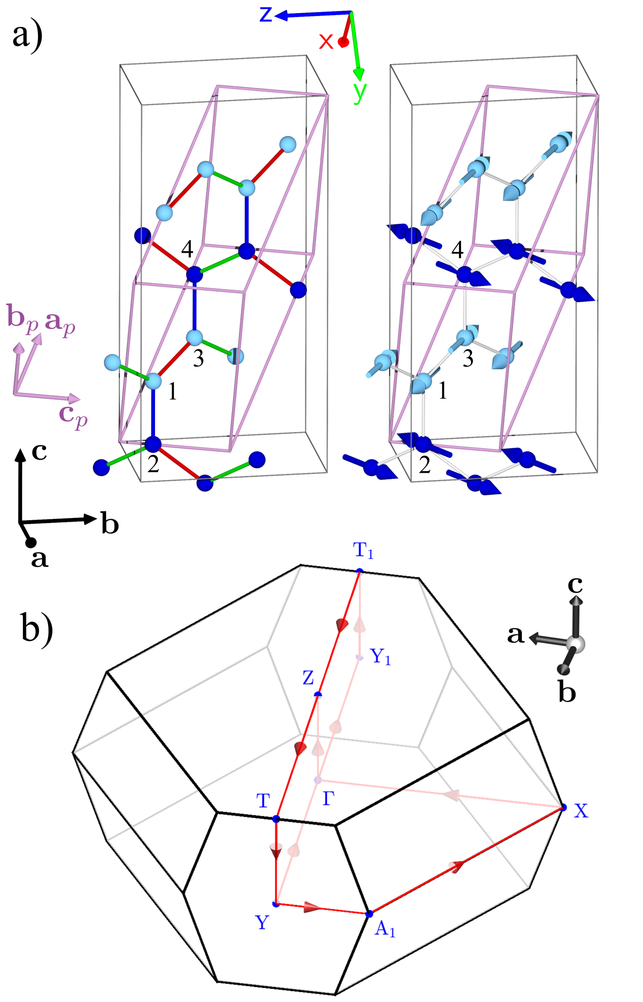

Supplementary Figure 9. **a,** Primitive (magenta) vs. conventional cell (thin black solid outline). Labels 1-4 show sites equivalent to those listed in Supplementary Table VI up to F-centring translations. **b,** Brillouin zone with ΓX, ΓY, ΓZ along the $-\mathbf{a}$, $\mathbf{b}$ and $\mathbf{c}$ axes, respectively. High-symmetry points connected by the thick red arrowed lines show the path along which the spinwave spectrum is plotted in Supplementary Fig. 10, which contains also the (hkl) indices of the labelled points.

and assuming an isotropic g-tensor in the ab plane). Such a large value of D/J is unphysical as D is typically a subleading exchange, arising from perturbative inclusion of the spin-orbit coupling. Furthermore, this JD Hamiltonian has rotational $U(1)$ symmetry around the z axis, so the moments in the ground state could be continuously rotated together in the ab plane around the z-axis at no energy cost via a gapless Goldstone mode, contrary to the observation of a clearly gapped magnetic spectrum in Fig. 4a. For those reasons we conclude that the $J\Gamma\Gamma'$ model discussed previously is a more likely minimal model consistent with all experimentally observed key features of the magnetic order and dynamics.

Supplementary Note 6. Spinwave spectrum

Primitive cell. Calculations of the spin-wave spectrum for model Hamiltonians were performed using SpinW [10] in the primitive magnetic cell, which coincides with the primitive structural cell, with basis vectors related to the conventional orthorhombic cell vectors by

$$\begin{pmatrix} \mathbf{a}_p \\ \mathbf{b}_p \\ \mathbf{c}_p \end{pmatrix} = \frac{1}{2} \begin{pmatrix} 0 & 1 & 1 \\ 1 & 0 & 1 \\ 1 & 1 & 0 \end{pmatrix} \begin{pmatrix} \mathbf{a} \\ \mathbf{b} \\ \mathbf{c} \end{pmatrix}.$$

There are four magnetic sublattices and six bonds per primitive cell, two each of type x, y and z, as illustrated in Supplementary Fig. 9a. For each of the two z-bonds the exchange matrix is $\mathcal{P}^T \mathcal{J}^{\mathsf{z}}_{abc} \mathcal{P}$, where $\mathcal{P}$ is the transformation matrix between the orthorhombic unit cell vectors and the $\mathfrak{X}\mathfrak{Y}\mathfrak{Z}$ SpinW Cartesian spin axes associated with the primitive cell,

$$\begin{pmatrix} \hat{\mathbf{a}} \\ \hat{\mathbf{b}} \\ \hat{\mathbf{c}} \end{pmatrix} = \mathcal{P} \begin{pmatrix} \hat{\mathfrak{X}} \\ \hat{\mathfrak{Y}} \\ \hat{\mathfrak{Z}} \end{pmatrix},$$

where $\hat{\mathfrak{X}} = \mathbf{a}_p/|\mathbf{a}_p|$, $\hat{\mathfrak{Z}} = \mathbf{a}_p \times \mathbf{b}_p/|\mathbf{a}_p \times \mathbf{b}_p|$ and $\hat{\mathfrak{Y}} = \hat{\mathfrak{Z}} \times \hat{\mathfrak{X}}$. The exchange matrix for the representative x-bond (1-3 on the $\mathbf{a}-\mathbf{b}$ chain in Fig. 9a is similarly obtained as $\mathcal{P}^T \mathcal{J}^{\mathsf{x}}_{abc} \mathcal{P}$ and the rest of the (four) exchange bonds in the primitive cell are obtained via symmetry operations of the crystal structure (2_y and 2_z rotations passing through the middle of the 3-4 z-bond).

Brillouin zone. The Brillouin zone corresponding to the primitive cell is illustrated in Supplementary Fig. 9b

and belongs to the F-centred orthorhombic unit cells with $1/a^2 > 1/b^2 + 1/c^2$ [11]. It has top and bottom distorted hexagonal faces with midpoints at $\pm(001)$, normal side distorted hexagonal faces with midpoints at $\pm(010)$ and additional eight slanted rectangular side faces with midpoints at $\left(\frac{1}{2}, \frac{1}{2}, \frac{1}{2}\right)$ and symmetry-equivalent positions obtained by mmm structural point group operations.

Key features of the spinwave spectrum. Four magnon modes are expected at a general wavevector, equal to the number of magnetic sublattices, but additional degeneracies occur in special cases. Starting with the simplest case $J = J' > 0$ and $\Gamma = \Gamma' = 0$, the ground state is collinear Néel ordered in an A basis vector with polarization selected via spontaneous symmetry-breaking with a linearly-dispersing gapless Goldstone mode emerging out of each Γ-point zone centre, as shown in the Supplementary Fig. 10a. At a general wavevector there are two doubly-degenerate magnon branches, with degeneracy protected by the rotational symmetry of the spin Hamiltonian.

Upon switching on an off-diagonal exchange $\Gamma > 0$ on the z-bonds the continuous rotational symmetry is broken and the ground state basis vector A_x is energetically selected, i.e. the magnetic structure remains collinear Néel ordered, but ordering breaks now a discrete Ising symmetry and as a consequence the spectrum has a gap above the magnetic Bragg peaks, scaling to leading order as $\sqrt{-\Gamma J}$. The breaking of rotational symmetry removes the two-fold degeneracy of the magnon branches with four non-degenerate modes at a general wavevector, as illustrated in the Supplementary Fig. 10b. The spectrum is identical between time-reversed domains, i.e. $\pm A_x$, and mirrored in h, k and l, as the spin Hamiltonian is invariant under reversal of any of x, y or z axes.

Upon additionally switching on a finite $\Gamma' > 0$ the structure becomes non-collinear by mixing into the ground state a $-C_y$ basis vector. The spectrum was already gaped with modes split, the addition of Γ' further increases the gap and also modifies the magnon energies such that the spectrum is no longer mirrored in k and time-reversed domains no longer have identical dispersion relations, as discussed in the following paragraph. A two-fold symmetry-protected degeneracy of the magnon bands is still preserved for the Brillouin zone boundary path $\mathrm{T_1ZT}$ as shown in Supplementary Fig. 10c.

Symmetry of the spinwave spectrum in reciprocal space. As discussed in Supplementary Note 3. the magnetic point group symmetry is $m'mm$ (m' normal to $\mathbf{b}$). Under operators of this point group a general wavevector $\mathbf{Q} = (hkl)$ is mapped into itself, or into $(\bar{h}k\bar{l})$, $(hk\bar{l})$ and $(\bar{h}kl)$. Therefore dispersion relations are mirrored in h and l, but not necessarily in k. Indeed this is shown in Supplementary Fig. 10c, note that the left-right symmetry of the dispersion relations along the paths $\mathrm{Y\Gamma Y_1}$ and $\mathrm{T_1ZT}$ are broken (the k-component of the reduced wavevector $\mathbf{q}$ switches sign in the middle of each of those two paths), i.e. left- and right-moving spin waves propagating along the (010) direction are non-reciprocal, with distinct dispersion relations. However, symmetry operations that are broken at the magnetic phase transition map one magnetic domain onto its time-reversed counterpart. The same operators map k into $\bar{k}$, therefore the dispersions of the time-reversed domain at wavevector $(h\bar{k}l)$ are the same as the dispersions of the original domain at (hkl), as apparent by comparing Supplementary Fig. 10c and d. The above mapping between the spectra of time-reversed domains has the consequence that for calculating the spherically averaged spectrum over wavevector orientations, relevant for comparison with the powder inelastic neutron scattering spectrum, it is sufficient to consider only one magnetic domain, as its time-reversed pair has an identical spherically-averaged spectrum. The non-reciprocal nature of the spinwave spectrum along (010) has the consequence that in a macroscopic single-crystal sample where both time-reversed magnetic domains co-exist, one would expect eight distinct spin wave modes (four for each of the two domains) at a general position in reciprocal space where the reduced wavevector has a finite k-component.

Transformation of the spinwave spectrum under sign reversal of the Γ' term. An important property of the Hamiltonian is that reversing the sign of Γ' is identical to reversing the y-axis, as can be seen by inspecting eqs. (5), (7) and (9). This has the consequence that reversing the sign of Γ' reverses the sign of the S_y components in the magnetic ground state, but the magnitude of the tilt angle ϕ and the ground state energy remain unchanged, see eqs. (10) and (11). Consider for concreteness the spectrum of the $\Gamma' > 0$ model for the $(A_x, -C_y)$ magnetic domain at wavevector (hkl). Reversal of the y-axis maps the Hamiltonian into the case of sign reversed Γ', the magnetic domain into (A_x, C_y) and the wavevector into $(h\bar{k}l)$. Applying now time reversal leaves the Hamiltonian invariant, maps the magnetic domain into its time-reversed counterpart $(-A_x, -C_y)$ and the wavevector into $(\bar{h}k\bar{l})$, which is equivalent to (hkl) via the magnetic point group operations. Therefore, the dispersions of the $\Gamma' > 0$ model and magnetic domain $(A_x, -C_y)$, and the sign reversed Γ' and magnetic domain $(-A_x, -C_y)$ are identical. The components of the dynamical correlations that contain one polarization along the y-axis, i.e. $S^{xy}(\mathbf{Q}, \omega)$, S^{yx}, S^{yz}, S^{zy} change sign, whereas all other components S^{xx}, S^{yy}, S^{zz}, S^{xz} and S^{zx} are unchanged, therefore the spherically-averaged spectrum is quite similar, but not identical between the two cases.

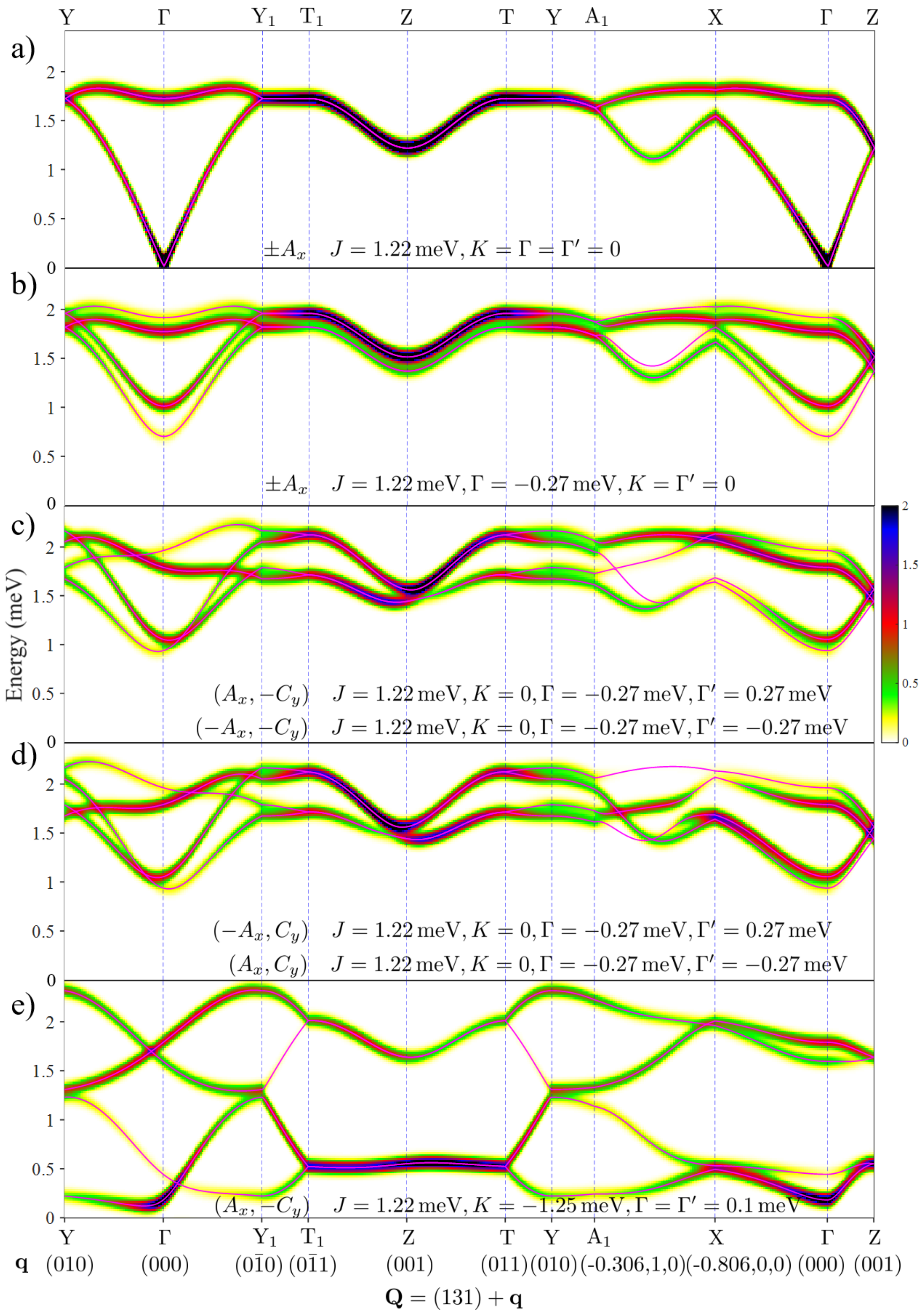

Supplementary Figure 10. Spinwave spectrum for various spin Hamiltonians discussed in the text, with magnetic domain and exchange parameters listed in each panel with $K' = K$ and $J' = J$. The wavevector follows the high-symmetry path in the Brillouin zone shown by the thick arrowed lines in Supplementary Fig. 9b, offset to the (131) zone centre chosen since almost all modes carry finite intensity. Horizontal top and bottom labels indicate special high-symmetry points in the Brillouin zone, also labelled in Supplementary Fig. 9b, and the indices show the reduced wavevector $\mathbf{q}$ in the Brillouin zone. Solid lines are the magnon dispersion relations and colour is the dynamical correlation $S^{zz}(\mathbf{Q}, \omega)$ after convolution with a Gaussian in energy of FWHM 0.1 meV (no magnetic form factor contribution). The powder-averaged spectrum for the top model in panels c and e is shown in Fig. 4b and Supplementary Fig. 8, respectively. The colour bar indicates the intensity in arbitrary units on a linear scale.

[1] R. Okuma, K. MacFarquharson, and R. Coldea, Selective synthesis and crystal chemistry of candidate rare earth Kitaev materials: honeycomb and hyperhoneycomb Na_2PrO_3, Inorg. Chem. **63**, 15941 (2024).

[2] R. Wolf and R. Hoppe, On Na_2PrO_3 and Na_2TbO_3, Z. Anorg. Allg. Chem. **556** (1988).

[3] A. Ramanathan, J. E. Leisen, and H. S. La Pierre, In-plane cation ordering and sodium displacements in layered honeycomb oxides with tetravalent lanthanides: Na_2LnO_3 (Ln = Ce, Pr, and Tb), Inorg. Chem. **60**, 1398 (2021).

[4] T. Takayama, A. Kato, R. Dinnebier, J. Nuss, H. Kono, L. Veiga, G. Fabbris, D. Haskel, and H. Takagi, Hyperhoneycomb iridate β-Li_2IrO_3 as a platform for Kitaev magnetism, Phys. Rev. Lett. **114**, 077202 (2015).

[5] G. M. Sheldrick, A short history of SHELX, Acta Cryst. A **64**, 112 (2008).

[6] A. Biffin, R. Johnson, S. Choi, F. Freund, S. Manni, A. Bombardi, P. Manuel, P. Gegenwart, and R. Coldea, Unconventional magnetic order on the hyperhoneycomb Kitaev lattice in β-Li_2IrO_3: Full solution via magnetic resonant x-ray diffraction, Phys. Rev. B **90**, 205116 (2014).

[7] H. T. Stokes, D. M. Hatch, and B. J. Campbell, Isotropy, https://iso.byu.edu/iso/isotropy.php.

[8] R. Okuma, K. MacFarquharson, R. D. Johnson, D. Voneshen, P. Manuel, and R. Coldea, Compass-model physics on the hyperhoneycomb lattice in the extreme spin-orbit regime: Data archive, Oxford University Research Archive Dataset (2024), https://doi.org/10.5287/ora-x582av5vx.

[9] E. K.-H. Lee and Y. B. Kim, Theory of magnetic phase diagrams in hyperhoneycomb and harmonic-honeycomb iridates, Phys. Rev. B **91**, 064407 (2015).

[10] S. Toth and B. Lake, Linear spin wave theory for single-Q incommensurate magnetic structures, J. Phys. Condens. Matter **27**, 166002 (2015).

[11] C. Bradley and A. Cracknell, *The Mathematical Theory of Symmetry in Solids* (Clarendon Press Oxford, 1972).
